# OLDER DYADS USING A WEARABLE PERSONAL SLEEP MONITORING DEVICE FOR SLEEP SELF-MANAGEMENT

**DOI:** 10.1093/geroni/igac059.2539

**Published:** 2022-12-20

**Authors:** Cynthia Jacelon, Maral Torrisan, Sarah Fiske

**Affiliations:** University of Massachusetts Amherst College of Nursing, Amherst, Massachusetts, United States; U. Massachusetts, Amherst, Amherst, Massachusetts, United States; Elaine Maarieb College of Nursing, University of Massachusetts Amherst, Amherst, Massachusetts, United States

## Abstract

Normally occurring changes in sleep patterns can affect behavior, safety, and function in older individuals. In addition, altered sleep of one partner can affect the functioning of the other. We tested a novel intervention using an off the shelf, wrist-worn actigraph as a personal sleep monitoring device (PSMD) with dyads comprised of individuals who were 70 years old or older, and slept in the same house. Aims included: 1) Establish the feasibility of sleep self-monitoring using PSMDs, as a self-management strategy. 2) Establish the feasibility of PSMD data sharing among members of the dyad to improve sleep self-management and improve sleep quality; and 3) Evaluate the usability of PSMDs and data sharing for dyads of older individuals. Over the course of a five-week trial, we used a mixed methods approach. Data were comprised of daily sleep diaries, data from the PSMD, weekly questionnaires regarding sleep patterns and function, and qualitative interviews focused on sleep, self-management within the dyad, and the usability of the PSMD. Data were analyzed using qualitative or statistical methods. The use of the PSMD increased awareness of sleep patterns at the individual and dyadic levels, but the limitations of the PSMD were frustrating. Participants valued having a graphic image of how their daily activities affected their sleep patterns. The dyadic approach was effective in improving sleep patterns for older individuals, and using over the counter activity monitoring devices provide a relatively inexpensive way to assist older adults to improve their sleep self-management.

